# Cytomegalovirus, Epstein-Barr virus and varicella zoster virus infection in the first two years of life: a cohort study in Bradford, UK

**DOI:** 10.1186/s12879-017-2319-7

**Published:** 2017-03-21

**Authors:** Lucy Pembrey, Dagmar Waiblinger, Paul Griffiths, Mauli Patel, Rafaq Azad, John Wright

**Affiliations:** 1 Department of Medical Statistics, London School of Hygiene and Tropical Medicine, Keppel Street, London, WC1E 7HT UK; 2 Bradford Institute for Health Research, Bradford, UK; 3 Centre for Virology, University College London Medical School, London, UK; 4 Virology Department, Royal Free Hospital, London, UK; 5 Department of Biochemistry, Bradford Royal Infirmary, Bradford, UK

**Keywords:** Cytomegalovirus, Epstein Barr virus, Varicella zoster virus, Children, Ethnic group, UK

## Abstract

**Background:**

Cytomegalovirus (CMV), Epstein Barr virus (EBV) and varicella-zoster virus (VZV) are common herpesviruses frequently acquired in childhood, which establish persistent, latent infection and are likely to impact the developing immune system. Little is known about the epidemiology of CMV and EBV infections in contemporary UK paediatric populations, particularly whether age at infection differs by ethnic group.

**Methods:**

Children enrolled in the Born in Bradford Allergy and Infection Study had a blood sample taken and a questionnaire completed at 12 and 24 months of age. Ordered logistic regression quantified associations between ethnicity and other risk factors and age at CMV/EBV/VZV infection (<12 months, 12–24 months, uninfected at 24 months).

**Results:**

Pakistani children (*n* = 472) were more likely to be infected with CMV and EBV at a younger age than White British children (*n* = 391) (CMV: adjusted odds ratio (OR) 2.53, 95% confidence interval (CI) 1.47–4.33; EBV: adjusted OR 2.16, 95% CI 1.43–3.26). Conversely, Pakistani children had lower odds of being VZV infected in the second year than White British children (adjusted OR 0.57, 95% CI 0.33–0.97). There was a strong association between increasing birth order and later CMV infection in Pakistani children.

**Conclusions:**

We report large differences in CMV and EBV incidence in the first 2 years between Pakistani and White British children born in Bradford, which cannot be explained by differences in risk factors for infection. Our data will inform the optimum schedule for future CMV and EBV vaccination programmes.

**Electronic supplementary material:**

The online version of this article (doi:10.1186/s12879-017-2319-7) contains supplementary material, which is available to authorized users.

## Background

Cytomegalovirus (CMV), Epstein Barr virus (EBV) and varicella-zoster virus (VZV) are common herpesviruses frequently acquired in childhood. They establish persistent, latent infection and are likely to impact the developing immune system [1, 2]. In childhood, VZV causes chickenpox but CMV and EBV infections are usually asymptomatic. If acquired in adolescence, EBV can cause glandular fever. EBV has been associated with immune disorders such as multiple sclerosis and lymphoma [3, 4] and CMV seropositivity is associated with inflammation, atherosclerosis and immunosenescence [5, 6].

Chickenpox vaccination is not part of the UK routine childhood vaccination programme but zoster vaccine has been introduced for adults in their 70s to prevent shingles [7], while CMV and EBV vaccines are under development [8–10]. Population-based data on the age-specific prevalence and incidence of these viruses is needed to inform the timing and target groups of potential vaccination programmes. Little is known about the epidemiology of CMV and EBV in contemporary paediatric UK populations, particularly in different ethnic groups. A recent US study identified differences in CMV seroprevalence by ethnic group among children but had limited data on other relevant variables such as childcare and breastfeeding [11].

The Allergy and Infection Study is a sub-study of the Born in Bradford birth cohort and aims to describe the maternal and paediatric epidemiology of CMV, EBV and VZV infection and to investigate the effect of age at infection on immune function and atopic allergy. The specific objectives of this paper are to estimate the incidence of CMV, EBV and VZV infection at age 1 and 2 years and to identify factors associated with infection, in particular to quantify any differences in age at infection by ethnic group.

## Methods

Children enrolled in the Born in Bradford cohort who were born on or after 1 March 2008 with a maternal baseline questionnaire available were eligible for the Allergy and Infection Study (ALL IN). Details of the Born in Bradford (BiB) cohort are described elsewhere [12]. Briefly, BiB is a multi-ethnic birth cohort study aiming to examine the impact of environmental, psychological and genetic factors on maternal and child health. Bradford is a city in the North of England with high levels of socio-economic deprivation and ethnic diversity.

Mothers were invited to participate in ALL IN 1 month before their child’s first birthday. A questionnaire was completed by those who consented and a 5 ml venous blood sample was taken from the child, centrifuged and stored at -80 °C. This was repeated 1 year later to provide exposure data and serum at 12 and 24 months. See Additional file 1 for further details.

A sample of 1000 children was selected for serological testing (see Additional file 1 for sample size calculations); 700 based on having 4 or 5 aliquots of the 24-month sample and having received the relevant immunisations (for a separate analysis of response to routine vaccination) and 300 randomly selected from those with fewer than 4 aliquots.

At the Royal Free Virology laboratory, the 24-month samples were tested first for CMV-immunoglobulin G (IgG), EBV-IgG and VZV-IgG. If positive, the 12-month sample was tested in the same way. CMV-IgG was tested using the Abbott Architect assay (kit product no. 6c15-25) and EBV-IgG and VZV-IgG using the Diasorin Liaison assays (kit product numbers 310510 and 310850, respectively) [13]. For children who were CMV-IgG positive at 12 months, the cord blood sample was tested for CMV- deoxyribonucleic acid (DNA) using a quantitative real-time polymerase chain reaction method described in detail elsewhere [14]. To verify the expected loss of maternal antibody by 12 months so that IgG testing at this age can represent paediatric infection, a random sample of 100 children who were IgG negative at 24 months for each infection were tested for IgG at 12 months.

### Data management and key variables

Data from several sources were combined: the maternal baseline questionnaire for the main BiB study, the maternity database (eClipse), the ALL IN questionnaire datasets (12- and 24-month) and the serology results.

Outcome variable: For each of the three infections, IgG results at 12 and 24 months were combined to create an age at infection variable for each child with three categories: infected by 12 months, infected between 12 and 24 months and uninfected at 24 months.

Explanatory variables: The main exposure of interest is ethnicity, defined as mother’s ethnic group and cultural background self-reported at the baseline interview during pregnancy, in three categories: White British, Pakistani and Other. Potential confounders are factors which influence exposure to infection via contact with the mother, siblings and others, especially children: birth order [15], breastfeeding [16, 17], household size/composition [15], childcare [18, 19], bedroom sharing [20, 21] and attendance at mother/baby activities [20]. We also considered socio-economic indicators (maternal education, home ownership) which may act as proxies for exposure to infection [11, 22].

Parents were asked for details of all regular childcare arrangements, defined as at least 5 h per week for a duration of at least 1 month. The hours at each childcare arrangement were calculated by: number of months attending the childcare arrangement (age 12 m questionnaire completed minus age started arrangement) x hours per week x 4.35 x weight for number of other children. The child-hours at each arrangement were added to obtain the total child care hours for each child. This was adapted from the method first used by Ma et al. [19]. Total child care hours were calculated separately for informal (looked after by family members/friends) and formal (childminder/nursery) arrangements. Data collected on breastfeeding at the 12- and 24-month visits were combined to create an ever/never breastfed variable and a breastfeeding duration variable.

Data on several variables were collected at more than one time point. Decisions were made *a priori* about which to include as follows: CMV—as most infections were acquired in the first year and the greatest difference between ethnic groups is in the first 12 months, childcare up to the 12-month visit was used, total number of members of household at baseline questionnaire was used rather than at 12-month visit, and the summary bedroom sharing variable used referred to the first 12 months. EBV—although there were differences by ethnic group in the first 12 months and from 12 to 24 months, most infections were acquired between 12 and 24 months. Household size and number of children in the household collected at the 12-month visit were used rather than the baseline questionnaire data, and bedroom sharing from 12 months collected at the 24-month visit was included. VZV—the difference between ethnic groups is at 12–24 months so the same variables were used as for the EBV analysis.

### Statistical methods

All data analysis was conducted using Stata version 13 [23].

To quantify the associations between age at CMV/EBV/VZV infection and risk factors for infection, ordered logistic regression was used, excluding 137 children who were not White British or Pakistani to focus the comparison between the two main groups. The resulting odds ratios are for infection by 12 months compared to infection between 12 and 24 months and infection between 12 and 24 months compared to uninfected at 24 months, i.e. the odds ratio compares each category with the next and the steps between categories are assumed to be equal. This proportional odds assumption was checked for each of the variables included. For the unstratified multivariable model of age at VZV infection, multinomial logistic regression was used as the proportional odds assumption was not satisfied.

First, multivariable models quantified the difference in age at infection between White British and Pakistani children, adjusting for other risk factors for infection. Due to differences in the prevalence of potential risk factors for infection between the White British and Pakistani children, analyses were then stratified by ethnic group to identify the most important risk factors in each group.

Most variables were included *a priori* as factors which influence exposure to infection. As some variables were likely to be highly correlated (e.g. birth order and number of children in household), likelihood ratio tests determined which of each pair should be retained in the model. Socio-economic factors were then added to the model and retained if the univariable *p* value was <0.05 or the likelihood ratio test *p* value for inclusion in the multivariable model was <0.05. Maternal age was included *a priori* in the CMV models as a proxy for maternal serostatus (CMV seropositivity increases with age). The timing of blood collection visits varied around 12 and 24 months of age; as the chance of a seropositive result would increase with age, the actual age at the planned 24-month blood sample variable was included in multivariable models.

## Results

**Fig. 1:**
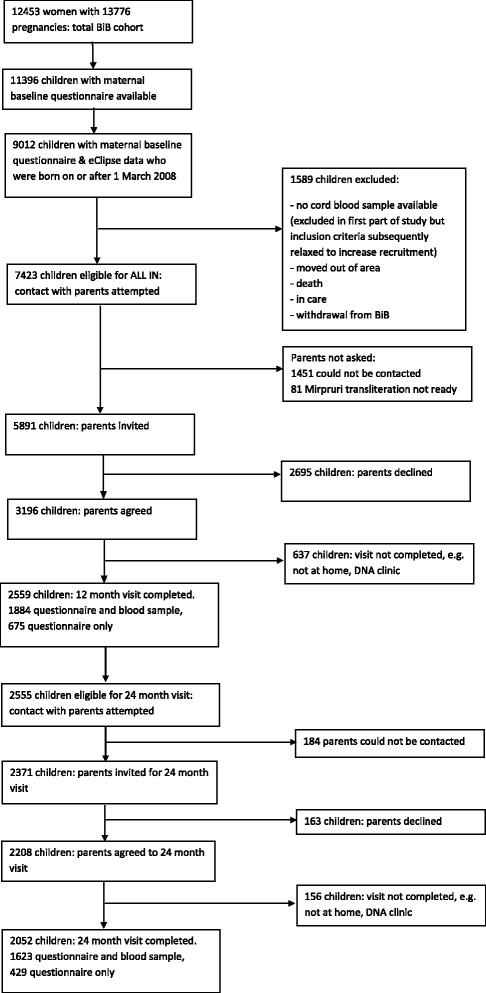
Children included in the Born in Bradford Allergy and Infection Study

Of 7423 eligible children, the parents of 5891 were contacted and invited to take part in ALL IN and 3196 (54%) agreed. Of the 2695 parents who declined, 2013 gave a reason: 1075 (53%) not happy about having blood samples taken, 510 (25%) too busy and 428 (21%) gave other reasons. Figure 1 shows the number of children completing a 12- and 24-month visit. The children recruited were born between March 2008 and June 2011.

Of the 24-month samples, there were 5 equivocal results for VZV which were considered negative. One-third of children (323) were seronegative for all three infections and 677 seropositive for at least one. A 12-month sample was not available for 58 of 677 children so 12-month samples for 619 children were tested. 231 children were CMV-IgG positive at 12 months. Cord blood was available for 154 of these; one child had detectable CMV-DNA (52,268 CMV VL genomes/ml) while 153 were undetectable (<200 copies/ml). Testing to verify loss of maternal antibody gave the following results: For EBV none of the 101 children who were IgG negative at 24 months were IgG positive at 12 months. For CMV two of 103 children, and for VZV two of 101 children, who were IgG negative at 24 months were IgG positive at 12 months (with the same results on re-testing).

This analysis includes 1000 children with CMV, EBV and VZV infection status at 12 and 24 months. 391 (39%) children were born to White British women, 472 (47%) to Pakistani women and 137 (14%) to women of other ethnic groups (Table 1). Table 1 shows the characteristics of the children and their mothers by ethnic group (also see Additional file 2).

**Table 1:** Characteristics of the children and their mothers

	All (*n* = 1000)	White British (*n* = 391)	Pakistani (*n* = 472)
Mother’s baseline questionnaire			
Mother’s ethnic group			
White British	391 (39%)		
White Other	26 (3%)		
Mixed-White and Black	8 (1%)		
Mixed-White and South Asian	5 (1%)		
Black	17 (2%)		
Indian	42 (4%)		
Pakistani	472 (47%)		
Bangladeshi	15 (1%)		
Other	24 (2%)		
Mother’s country of birth			
UK & Ireland	612 (61%)	386 (99%)	177 (38%)
Elsewhere	388 (39%)	5 (1%)	295 (62%)
Mother’s age at recruitment (yrs) mean, SD, range			
	28.9, 5.57, 15–44	28.9, 5.96, 15–43	28.8, 5.28, 17–44
Marital & cohabitation status combined			
Married and living with partner	716 (72%)	167 (43%)	445 (94%)
Not married and living with partner	170 (17%)	146 (37%)	1
Not living with partner	113 (11%)	77 (20%)	26 (6%)
missing (married, cohab status missing)	1	1	0
Birth order			
1	367 (37%)	184 (47%)	126 (27%)
2	289 (29%)	128 (33%)	107 (23%)
3–4	290 (29%)	72 (18%)	194 (41%)
5+	54 (5%)	7 (2%)	45 (9%)
Total no. of household members			
median, IQR, range	4, 3–5, 1–16	3, 2–3, 1–8	5, 4–7, 2–16
missing	1	1	0
No. of household members under 16 years			
median, IQR, range	1, 0–2, 0–7	1, 0–1, 0–6	2, 1–3, 0–7
missing	1	1	0
Mother’s highest educational qualification (equivalised)			
< 5 GCSE equivalent	200 (20%)	59 (15%)	130 (28%)
5 GCSE equivalent	253 (25%)	98 (25%)	135 (29%)
A level equivalent	147 (15%)	72 (18%)	55 (12%)
higher than A level	313 (31%)	118 (30%)	134 (28%)
Other/don’t know/foreign unknown	86 (9%)	44 (11%)	17 (3%)
missing	1	0	1
Mother’s employment status			
Currently employed	461 (46%)	279 (71%)	94 (20%)
Previously employed	238 (24%)	91 (23%)	117 (25%)
Never employed	300 (30%)	21 (5%)	261 (55%)
missing	1		
Father’s employment status			
Employed non-manual	414 (44%)	215 (59%)	135 (30%)
Employed manual	298 (31%)	85 (23%)	172 (38%)
Self-employed	166 (17%)	38 (10%)	111 (25%)
Student	9 (1%)	4 (1%)	2
Unemployed	54 (6%)	21 (6%)	28 (6%)
Don’t know	5 (1%)	4 (1%)	1
missing	54	24	23
Mother smoked during pregnancy			
Yes	135 (14%)	110 (28%)	15 (3%)
No	863 (86%)	281 (72%)	456 (97%)
missing	2		1
Index of Multiple Deprivation (IMD) 2010 quintile (national)			
1 (most deprived)	640 (64%)	162 (41%)	387 (82%)
2	164 (16%)	87 (22%)	49 (10%)
3	141 (14%)	94 (24%)	30 (6%)
4	35 (4%)	31 (8%)	4 (1%)
5 (least deprived)	20 (2%)	17 (4%)	2
Maternity records			
Parity (registerable) median, IQR, range			
	1, 0–2, 0–7	1, 0–1, 0–6	2, 0–3, 0–7
missing	26	10	14
Gestational age (to last completed week) median, IQR, range			
	39, 38–40, 29–44	40, 38–40, 29–44	39, 38–40, 20–42
missing	6	2	3
Birth weight (g) median, IQR, range			
	3280, 2920–3610, 890–4904	3400, 3050–3710, 890–4820	3180, 2880–3490, 1275–4520
missing	6	2	3
Route of birth			
vaginal	783 (79%)	296 (76%)	387 (83%)
caesarean	211 (21%)	93 (24%)	82 (17%)
missing	6	2	3
Sex of baby			
Male	531 (53%)	190 (49%)	262 (55%)
Female	469 (47%)	201 (51%)	210 (44%)
ALL IN questionnaires			
Ever breastfed	806 (81%)	289 (74%)	386 (82%)
Never breastfed	192 (19%)	102 (26%)	84 (18%)
missing	2	0	2
Duration of breastfeeding			
never	192 (19%)	102 (26%)	84 (18%)
< =4 weeks	210 (21%)	99 (25%)	91 (19%)
1–6 months	243 (24%)	95 (24%)	121 (26%)
6–12 months	219 (22%)	65 (17%)	102 (22%)
> 12 months	131 (13%)	29 (7%)	70 (15%)
missing	5	1	4
Bedroom/bed sharing			
Shared bedroom up to 6 mths: Yes	912 (92%)	323 (83%)	463 (99%)
No	82	66	5
missing	6	2	4
Shared bedroom from 6 mths to 12mth visit: Yes	716 (72%)	170 (44%)	439 (94%)
No	278	219	29
missing	6	2	4
Shared bedroom from 12 mths: Yes	688 (69%)	143 (37%)	448 (95%)
No	312	248	24
Shared bed from 12 mths: Yes	273 (27%)	27 (7%)	204 (43%)
No	726	364	267
missing	1		1
Childcare			
Ever regular childcare by 12 m visit	348 (35%)	228 (58%)	63 (13%)
Never regular childcare by 12 m	646	161	405
missing	6	2	4
Ever regular childcare by 24 m visit	426 (43%)	266 (68%)	92 (19%)
Never regular childcare by 24 m	574 (57%)	125 (32%)	380 (81%)
Mother & baby activities up to 6 m			
Rarely	634 (64%)	169 (44%)	383 (82%)
At least once a month	41 (4%)	24 (6%)	11 (2%)
Usually once a week	191 (19%)	104 (27%)	55 (12%)
More than once a week	126 (13%)	91 (23%)	18 (4%)
missing	8	3	5
Mother & baby activities 6 m to 12 m visit			
Rarely	593 (60%)	175 (45%)	337 (72%)
At least once a month	66 (7%)	32 (8%)	26 (6%)
Usually once a week	203 (20%)	92 (24%)	79 (17%)
More than once a week	132 (13%)	90 (23%)	26 (5%)
missing	6	2	4
Ever travelled outside UK up to 12 m visit			
No	773 (78%)	320 (82%)	373 (79%)
Within Europe	93 (9%)	61 (16%)	4 (1%)
Outside Europe	128 (13%)	7 (2%)	92 (20%)
missing	6	3	3
Travelled outside UK since 1st birthday (up to 24 m visit)			
No	686 (69%)	271 (69%)	343 (73%)
Within Europe	155 (15%)	110 (28%)	12 (2%)
Outside Europe	159 (16%)	10 (3%)	117 (25%)

The 1000 children selected for this analysis were similar to the ALL IN children who were not included with respect to ethnic group, mother’s country of birth, sex, childcare, household size and number of children in the household but were more likely to have been breastfed (81% vs. 74%). Compared to the rest of the BiB cohort (12,538 children), the 1000 ALL IN children were similar in terms of most characteristics but their mothers were slightly more likely to be married and living with partner (72% vs. 65%) and to be in the least deprived quintiles of the Index of Multiple Deprivation.

**Fig. 2:**
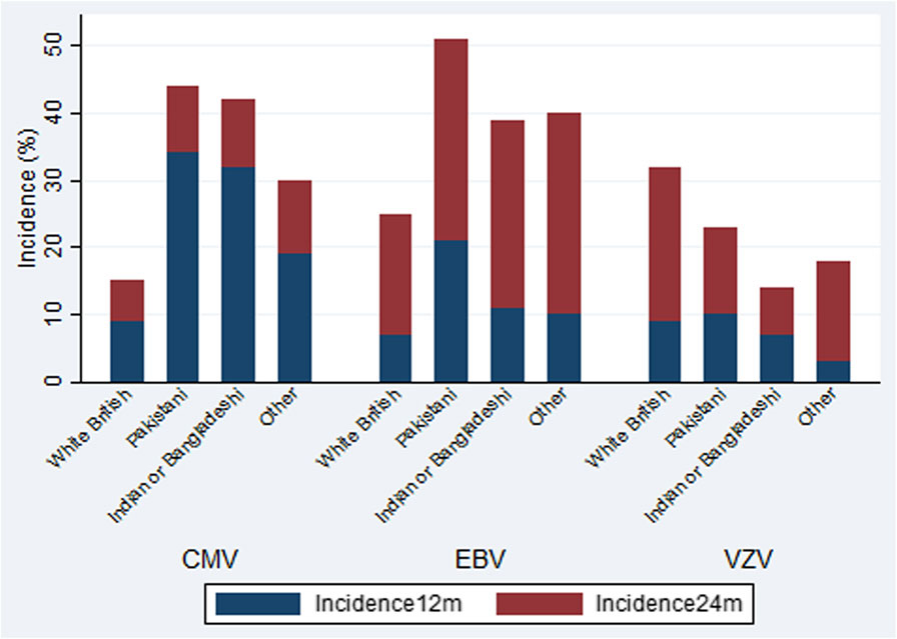
Incidence of CMV, EBV and VZV infection at 12 and 24 months by ethnic group

There were marked differences in the incidence of CMV and EBV infection at 12 and 24 months between the White British and Pakistani children with the Pakistani children infected earlier (Fig. 2 and Additional file 3).

### CMV

One-third of Pakistani children (162, 34%) were infected by 12 months compared to 9% (36) of White British children, with a further 10% (47) and 6% (25) respectively infected between 12 and 24 months. In unadjusted analysis there was a 4-fold increase in odds of being infected by 12 months than between 12 and 24 months or infected between 12 and 24 months than uninfected at 24 months in Pakistani children compared to White British children (odds ratio (OR) 4.44, 95% confidence interval (CI) 3.20–6.14, <0.001). This association attenuated with adjustment for covariates (birth order, household size, breastfeeding duration, bedroom sharing, childcare, mother/baby activities, travel outside Europe, maternal age, mother’s country of birth, home ownership and age blood sample taken) but there remained strong evidence of a 2.5-fold increase in odds of earlier infection among Pakistani children compared to White British children (adjusted OR = 2.53, 95% CI 1.47–4.33, *p* = 0.001).

Maternal infection status was only available for 90 children; 71/90 (79%) mothers were CMV seropositive (52% (17/33) of White British and 94% (48/51) of Pakistani women). Among the children of seropositive mothers, 3/17 (18%) White British children were infected by 12 months compared to 14/48 (29%) Pakistani children. An adjusted sub-group analysis was not possible due to small numbers in some groups but the pattern of earlier infection among Pakistani children remains.

To identify the most important risk factors in each group, the adjusted analysis was run separately for White British and Pakistani children (Table 2). The key associations are summarised in Table 5. Of particular note, among the Pakistani children increasing duration of breastfeeding was strongly associated with earlier infection whereas among White British children earlier infection was only evident for those breastfed for 6 months or more. There was a strong association between increasing birth order and later age at infection in the Pakistani children such that children with one or more older siblings had 60% lower odds of being infected by 12 months or between 12 and 24 months than first-born children. The birth order estimates for White British children were in the same direction but no statistically significant association was observed.

**Table 2:** Unadjusted and adjusted odds ratios for the association between age at CMV infection and exposure variables, stratified by ethnic group

White British		Age at CMV infection			Unadjusted OR (95% CI)	Unadjusted OR (95% CI) *n* = 379	Adjusted OR (95% CI) *n* = 379
		by 12 mths	12–24 mths	uninfected at 24 mths			
All children, *n* = 391		36 (9%)	25 (6%)	330 (84%)			
Birth order (391)	1	20 (11%)	7 (4%)	157 (85%)	1.0	1.0	1.0
	2	11 (9%)	12 (9%)	105 (82%)	1.21 (0.66–2.22)	1.19 (0.65–2.18)	0.81 (0.38–1.70)
	3+	5 (6%)	6 (8%)	68 (86%)	0.90 (0.42–1.91)	0.80 (0.37–1.74)	0.47 (0.14–1.52)
Total household members at baseline (390)	median IQR, range	3 2–3.5, 2–8	3 2–4, 1–6	3 2–3, 1–8	1.15 (0.91–1.46)	1.16 (0.91–1.49)	1.42 (1.01–1.99)
Duration of breastfeeding (390)	never	7 (7%)	5 (5%)	90 (88%)	1.0	1.0	1.0
	<=4 weeks	7 (7%)	7 (7%)	85 (86%)	1.22 (0.53–2.78)	1.10 (0.48–2.55)	1.16 (0.47–2.82)
	1–6 months	4 (4%)	5 (5%)	86 (91%)	0.77 (0.31–1.93)	0.78 (0.31–1.93)	0.89 (0.33–2.40)
	6–12 months	12 (19%)	8 (12%)	45 (69%)	3.29 (1.49–7.28)	3.20 (1.45–7.10)	4.03 (1.54–10.52)
	>12 months	6 (21%)	0	23 (79%)	2.18 (0.74–6.45)	2.08 (0.70–6.16)	4.05 (1.19–13.78)
Bedroom sharing in first year (389)	no	18 (8%)	14 (6%)	188 (85%)	1.0	1.0	1.0
	yes	18 (11%)	11 (6%)	140 (83%)	1.23 (0.71–2.12)	1.22 (0.70–2.11)	1.67 (0.85–3.26)
Formal child care up to 12 m visit (381)	No childcare	13 (8%)	6 (4%)	142 (88%)	1.0	1.0	1.0
	<1165 total hours	3 (9%)	0	31 (91%)	0.74 (0.21–2.67)	0.74 (0.21–2.65)	0.90 (0.23–3.57)
	> = 1165 total hours	11 (12%)	16 (17%)	68 (71%)	2.69 (1.41–5.15)	2.71 (1.42–5.18)	3.63 (1.61–8.17)
	No formal (only informal)	9 (9%)	2 (2%)	80 (88%)	1.05 (0.47–2.31)	1.04 (0.47–2.29)	1.39 (0.59–3.32)
Informal child care up to 12 m visit (376)	No childcare	13 (8%)	6 (4%)	142 (88%)	1.0	-	-
	<1165 total hours	10 (8%)	5 (4%)	104 (87%)	1.08 (0.52–2.22)		
	> = 1165 total hours	3 (20%)	0	12 (80%)	2.05 (0.53–7.96)		
	No informal (only formal)	8 (9%)	14 (17%)	59 (73%)	2.52 (1.28–4.96)		
Mother and baby activities in first year (389)	Rarely	22 (11%)	13 (6%)	174 (83%)	1.0	1.0	1.0
	At least once a month	14 (8%)	12 (7%)	154 (86%)	0.83 (0.48–1.43)	0.87 (0.50–1.51)	0.74 (0.39–1.40)
Maternal age at recruitment (391)	Mean, SD, range	27.9, 6.1 15–38	30.1, 5.7 18–43	29.0, 6.0 16–43	0.99 (0.95–1.04)	0.99 (0.95–1.04)	0.99 (0.93–1.05)
Home ownership (391)	Yes	20 (8%)	15 (6%)	210 (86%)	1.0	1.0	1.0
	No	16 (11%)	10 (7%)	120 (82%)	1.31 (0.75–2.27)	1.31 (0.75–2.28)	2.07 (0.99–4.35)
Age 24 m blood sample taken (mths) (391)	23.59–24.99	6 (7%)	3 (4%)	75 (89%)	1.0	1.0	1.0
	25–26.99	23 (10%)	19 (8%)	186 (82%)	1.84 (0.86–3.96)	2.11 (0.95–4.71)	1.60 (0.68–3.77)
	27–32.89	7 (9%)	3 (4%)	69 (87%)	1.22 (0.47–3.17)	1.38 (0.51–3.69)	0.95 (0.33–2.76)
Pakistani		by 12 mths	12–24 mths	uninfected at 24 mths	Unadjusted OR (95% CI)	Unadjusted OR (95% CI) *n* = 466	Adjusted OR (95% CI) *n* = 466
All children, *n* = 472		162 (34%)	47 (10%)	263 (56%)			
Birth order (472)	1	63 (50%)	14 (11%)	49 (39%)	1.0	1.0	1.0
	2	31 (29%)	13 (12%)	63 (59%)	0.43 (0.26–0.72)	0.43 (0.26–0.71)	0.41 (0.23–0.73)
	3+	68 (29%)	20 (8%)	151 (63%)	0.38 (0.25–0.58)	0.37 (0.24–0.56)	0.37 (0.22–0.64)
Total household members at baseline (472)	median IQR, range	5 4–7, 2–12	5 4–7, 2–16	5 4–6, 2–12	1.09 (1.01–1.18)	1.09 (1.01–1.17)	1.08 (0.99–1.17)
Duration of breastfeeding (468)	never	7 (8%)	7 (8%)	70 (84%)	1.0	1.0	1.0
	<=4 weeks	20 (22%)	10 (11%)	61 (67%)	2.49 (1.22–5.08)	2.53 (1.24–5.17)	2.60 (1.22–5.53)
	1–6 months	45 (37%)	7 (6%)	69 (57%)	4.27 (2.18–8.36)	4.27 (2.18–8.36)	3.61 (1.79–7.27)
	6–12 months	51 (50%)	12 (12%)	39 (38%)	8.34 (4.21–16.51)	8.53 (4.30–16.92)	7.38 (3.61–15.10)
	>12 months	37 (53%)	11 (16%)	22 (31%)	10.03 (4.85–20.75)	10.04 (4.85–20.77)	12.57 (5.81–27.19)
Bedroom sharing in the first year (468)	no	8 (26%)	2 (6%)	21 (68%)	1.0	1.0	1.0
	yes	153 (35%)	45 (10%)	239 (55%)	1.68 (0.78–3.63)	1.68 (0.78–3.62)	1.60 (0.69–3.71)
Any regular child care arrangement up to 12 m visit (468)	no	139 (34%)	39 (10%)	227 (56%)	1.0	1.0	1.0
	yes	22 (35%)	8 (13%)	33 (52%)	1.10 (0.66–1.84)	1.14 (0.68–1.91)	1.23 (0.67–2.25)
Mother and baby activities in the first year (468)	Rarely	146 (36%)	40 (9%)	219 (54%)	1.0	1.0	1.0
	At least once a month	15 (24%)	7 (11%)	41 (65%)	0.61 (0.35–1.05)	0.61 (0.35–1.05)	0.65 (0.35–1.19)
Travel outside Europe by 12 m visit (469)	No	134 (36%)	40 (11%)	203 (54%)	1.0	1.0	1.0
	yes	27 (29%)	7 (7%)	58 (63%)	0.71 (0.45–1.12)	0.72 (0.45–1.14)	0.52 (0.31–0.87)
Maternal age at recruitment (472)	Mean, SD, range	28.2, 5.02 17–42	27.6, 4.19 19–36	29.3, 5.55 17–44	0.96 (0.93–0.99)	0.96 (0.92–0.99)	0.99 (0.94–1.03)
Mother’s country of birth (472)	UK or Ireland	47 (26%)	19 (11%)	111 (63%)	1.0	1.0	1.0
	Elsewhere	115 (39%)	28 (9%)	152 (52%)	1.64 (1.13–2.38)	1.68 (1.15–2.44)	1.87 (1.20–2.92)
Home ownership (472)	Yes	94 (31%)	30 (10%)	183 (59%)	1.0	1.0	1.0
	No	68 (41%)	17 (10%)	80 (49%)	1.58 (1.09–2.28)	1.56 (1.08–2.26)	1.27 (0.83–1.94)
Age 24 m blood sample taken (mths) (472)	23.59–24.99	27 (30%)	6 (7%)	58 (64%)	1.0	1.0	1.0
	25–26.99	88 (37%)	29 (12%)	123 (51%)	1.56 (0.96–2.54)	1.50 (0.92–2.45)	1.22 (0.71–2.12)
	27–32.89	47 (33%)	12 (9%)	82 (58%)	1.24 (0.73–2.12)	1.19 (0.70–2.04)	0.91 (0.49–1.69)

*CMV* cytomegalovirus; *OR* odds ratio; *CI* confidence interval; *IQR* inter-quartile range

### EBV

7% (28) of White British children were infected by 12 months compared to 21% (101) of Pakistani children and a further 18% (71) and 30% (140) respectively were infected between 12 and 24 months. In unadjusted ordered logistic regression analysis, Pakistani children had three times the odds of being EBV infected by 12 months than between 12 and 24 months or between 12 and 24 months than to remain uninfected at 24 months than the White British children (OR 3.14, 95% CI 2.36–4.18, *p* <0.001). When adjusting for birth order, household size at 12 months, bedroom sharing in the first year, breastfeeding duration, formal childcare hours in the first and second years, mother/baby activities in the first year, travel outside Europe since first birthday, maternal age, maternal education and age blood sample taken, Pakistani children had twice the odds of being infected earlier than White British children (adjusted OR 2.16, 95% CI 1.43–3.26, *p* < 0.001).

The analysis was stratified by ethnic group (Table 3), with key associations summarised in Table 5. The unexpected strong association between later infection and mother/baby activities is limited to White British children who had ever attended childcare and relates to attendance between 6 and 12 months (rather than <6 months) but there was no clear dose-response relationship with the original 4-category variable. In multivariable analysis among White British children, the odds ratios for the original variable for mother/baby activities between 6 and 12 months were around 0.5 but only one was of (borderline) statistical significance (*p* = 0.05).

**Table 3:** Unadjusted and adjusted odds ratios for the association between age at EBV infection and exposure variables, stratified by ethnic group

White British		Age at EBV infection			Unadjusted OR (95% CI)	Unadjusted OR (95% CI) *n* = 361	Adjusted OR (95% CI) *n* = 361
		by 12 mths	12–24 mths	uninfected at 24 mths			
All children, *n* = 391		28 (7%)	71 (18%)	292 (75%)			
Birth order (391)	1	10 (5%)	33 (18%)	141 (77%)	1.0	-	-
	2	10 (8%)	18 (14%)	100 (78%)	0.95 (0.56–1.63)	-	-
	3+	8 (10%)	20 (25%)	51 (65%)	1.80 (1.02–3.17)	-	-
Total household members at 12 m (389)	median IQR, range	4 3–4.5, 2–8	4 3–5, 2–7	4 3–4, 2–8	1.20 (0.97–1.47)	1.19 (0.97–1.47)	0.91 (0.63–1.33)
No. of children (<16y) in household at 12 m (369)	median IQR, range	2 1–3, 1–6	1 1–2, 1–5	2 1–2, 1–4	1.36 (1.07–1.74)	1.34 (1.05–1.71)	1.53 (0.96–2.42)
Duration of breastfeeding (390)	never	12 (12%)	20 (19%)	70 (69%)	1.0	1.0	1.0
	<=4 weeks	8 (8%)	20 (20%)	71 (72%)	0.83 (0.46–1.51)	0.78 (0.42–1.47)	0.96 (0.48–1.89)
	1–6 months	7 (7%)	16 (17%)	72 (76%)	0.68 (0.36–1.27)	0.62 (0.33–1.18)	0.88 (0.43–1.78)
	6–12 months	1 (2%)	11 (17%)	53 (81%)	0.46 (0.22–0.97)	0.44 (0.21–0.94)	0.74 (0.31–1.76)
	>12 months	0	4 (14%)	25 (86%)	0.32 (0.10–1.00)	0.31 (0.10–0.96)	0.58 (0.17–2.00)
Bedroom sharing in first year (389)	no	15 (7%)	36 (16%)	169 (77%)	1.0		-
	yes	13 (8%)	35 (21%)	121 (72%)	1.30 (0.82–2.04)		-
Bedroom sharing in second year (391)	No	14 (6%)	43 (17%)	191 (77%)	1.0	1.0	1.0
	yes	14 (10%)	28 (19%)	101 (71%)	1.43 (0.90–2.27)	1.59 (0.99–2.57)	1.21 (0.71–2.07)
Informal child care from 12 m to 24 m visit: total hours (384)	No childcare	12 (10%)	21 (17%)	92 (74%)	1.0	1.0	1.0
	<1165 total hours	3 (7%)	13 (29%)	28 (64%)	1.45 (0.71–2.96)	1.31 (0.62–2.78)	2.48 (1.04–5.92)
	> = 1165 total hours	4 (10%)	6 (15%)	31 (75%)	0.91 (0.40–2.05)	0.89 (0.39–2.02)	1.54 (0.61–3.86)
	No informal (only formal)	3 (3%)	17 (16%)	87 (81%)	0.61 (0.33–1.13)	0.56 (0.30–1.05)	0.87 (0.43–1.75)
	No 24 m childcare, only 12 m	6 (9%)	12 (18%)	49 (73%)	1.01 (0.52–1.97)	1.00 (0.51–1.99)	1.05 (0.50–2.20)
Mother and baby activities in first year (389)	Rarely	23 (11%)	44 (21%)	142 (68%)	1.0	1.0	1.0
	At least once a month	5 (3%)	27 (15%)	148 (82%)	0.44 (0.27–0.71)	0.44 (0.27–0.71)	0.53 (0.31–0.90)
Mother and baby activities in second year (389)	Rarely	13 (8%)	34 (22%)	111 (70%)	1.0		-
	At least once a month	15 (6%)	37 (16%)	179 (78%)	0.69 (0.44–1.09)		-
Maternal age at recruitment (391)	Mean, SD, range	26.6, 5.34 18–35	28.1, 5.83 16–41	29.4, 5.99 15–43	0.95 (0.91–0.99)	0.95 (0.91–0.99)	0.96 (0.91–1.01)
Maternal education (391)	5 GCSE equivalent or less	18 (11%)	32 (20%)	107 (68%)	1.0	1.0	1.0
	A level equiv & higher	9 (5%)	32 (17%)	149 (78%)	0.56 (0.35–0.91)	0.55 (0.34–0.89)	0.86 (0.47–1.60)
	other/DK/foreign unknown	1 (2%)	7 (16%)	36 (82%)	0.45 (0.20–1.03)	0.36 (0.14–0.91)	0.46 (0.17–1.25)
Home ownership (391)	Yes	12 (5%)	41 (17%)	192 (78%)	1.0	1.0	1.0
	No	16 (11%)	30 (21%)	100 (68%)	1.73 (1.09–2.73)	1.65 (1.03–2.64)	0.94 (0.50–1.74)
Age 24 m blood sample taken (mths) (391)	23.59–24.99	9 (11%)	11 (13%)	64 (76%)	1.0	1.0	1.0
	25–26.99	18 (8%)	36 (16%)	174 (76%)	0.96 (0.53–1.72)	1.01 (0.55–1.87)	1.04 (0.54–1.99)
	27–32.89	1 (1%)	24 (30%)	54 (68%)	1.26 (0.64–2.48)	1.26 (0.62–2.57)	1.21 (0.57–2.57)
Pakistani		by 12 mths	12–24 mths	uninfected at 24 mths	Unadjusted OR (95% CI)	Unadjusted OR (95% CI) *n* = 453	Adjusted OR (95% CI) *n* = 453
All children, *n* = 472		101 (21%)	140 (30%)	231 (49%)			
Birth order (472)	1	26 (21%)	38 (30%)	62 (49%)	1.0		-
	2	24 (22%)	31 (29%)	52 (49%)	1.05 (0.65–1.71)		-
	3+	51 (21%)	71 (30%)	117 (49%)	1.02 (0.68–1.53)		-
Total household members at 12 m (468)	median IQR, range	6 5–7, 3–13	6 4–7, 3–15	5 4–7, 2–15	1.07 (1.00–1.16)	1.07 (0.99–1.15)	1.07 (0.98–1.17)
No. of children (<16y) in household at 12 m (456)	median IQR, range	3 2–4, 1–8	3 2–4, 1–7	3 2–4, 1–7	1.08 (0.95–1.23)	1.08 (0.94–1.22)	1.03 (0.87–1.20)
Duration of breastfeeding (468)	never	21 (25%)	21 (25%)	42 (50%)	1.0	1.0	1.0
	<=4 weeks	25 (27%)	29 (32%)	37 (41%)	1.34 (0.77–2.35)	1.53 (0.87–2.70)	1.65 (0.92–2.96)
	1–6 months	24 (20%)	36 (30%)	61 (50%)	0.90 (0.53–1.53)	0.87 (0.51–1.49)	0.82 (0.48–1.43)
	6–12 months	17 (17%)	32 (31%)	53 (52%)	0.82 (0.47–1.41)	0.82 (0.47–1.43)	0.73 (0.41–1.29)
	>12 months	14 (20%)	19 (27%)	37 (53%)	0.84 (0.46–1.54)	0.87 (0.47–1.61)	0.88 (0.47–1.63)
Bedroom sharing in first year (468)	no	2 (6%)	10 (32%)	19 (61%)	1.0	1.0	1.0
	yes	99 (23%)	128 (29%)	210 (48%)	1.94 (0.95–3.96)	1.95 (0.95–3.99)	2.06 (0.99–4.28)
Bedroom sharing in second year (472)	no	4 (17%)	5 (21%)	15 (62%)	1.0		-
	yes	97 (22%)	135 (30%)	216 (48%)	1.70 (0.74–3.90)		-
Bed sharing in second year (471)	No	55 (21%)	70 (26%)	142 (53%)	1.0		-
	yes	46 (22%)	69 (34%)	89 (44%)	1.35 (0.96–1.90)		-
Any regular child care arrangement up to 24 m visit (472)	no	87 (23%)	106 (28%)	187 (49%)	1.0	1.0	1.0
	yes	14 (15%)	34 (37%)	44 (48%)	0.91 (0.60–1.38)	0.89 (0.58–1.37)	0.97 (0.62–1.53)
Mother and baby activities in first year (468)	Rarely	88 (22%)	124 (31%)	193 (48%)	1.0	1.0	1.0
	At least once a month	13 (21%)	14 (22%)	36 (57%)	0.74 (0.44–1.25)	0.67 (0.40–1.15)	0.61 (0.35–1.06)
Mother and baby activities in second year (472)	Rarely	58 (23%)	78 (31%)	117 (46%)	1.0		-
	At least once a month	43 (20%)	62 (28%)	114 (52%)	0.80 (0.57–1.13)		-
Age 24 m blood sample taken (mths) (472)	23.59–24.99	20 (22%)	22 (24%)	49 (54%)	1.0	1.0	1.0
	25–26.99	46 (19%)	70 (29%)	124 (52%)	1.02 (0.64–1.62)	0.98 (0.61–1.57)	1.10 (0.67–1.80)
	27–32.89	35 (25%)	48 (34%)	58 (41%)	1.50 (0.91–2.47)	1.47 (0.88–2.46)	1.78 (1.05–3.04)

*EBV* Epstein-Barr virus; *OR* odds ratio; *CI* confidence interval; *IQR* inter-quartile range

### VZV

The incidence of VZV infection at 12 months was similar for Pakistani and White British children (10% and 9% respectively) but between 12 and 24 months incidence was higher in the White British children (23% vs. 14% in the Pakistani children). In unadjusted multinomial logistic regression analysis, there was no difference in the proportion of children infected by 12 months by ethnic group (OR 1.00, 95% CI 0.62–1.60) but Pakistani children had nearly 50% lower odds of being infected between 12 and 24 months than White British children (OR 0.53, 95% CI 0.37–0.76, *p* = 0.001). This association remained, although with wider confidence intervals, after adjusting for birth order, household size at 12 months, breastfeeding duration, formal childcare hours in first and second years, bedroom sharing in the second year, mother/baby activities in the second year, maternal age and age blood sample taken: by 12 m adjusted OR 0.83, 95% CI 0.41–1.68, *p* = 0.60; 12–24 m adjusted OR 0.57, 95% CI 0.33–0.97, *p* = 0.04.

Results of the multivariable ordered logistic regression analysis, stratified by ethnic group, are shown in Table 4. Higher birth order was associated with earlier VZV infection in White British and Pakistani children, with second-born children having the highest odds of earlier infection. Among the Pakistani children, for every year increase in maternal age there was a 5% decrease in odds of early VZV infection (Table 4).

**Table 4:** Unadjusted and adjusted odds ratios for the association between age at VZV infection and exposure variables, stratified by ethnic group

White British		Age at VZV infection			Unadjusted OR (95% CI)	Unadjusted OR (95% CI) *n* = 378	Adjusted OR (95% CI) *n* = 378
		by 12 mths	12–24 mths	uninfected at 24 mths			
All children, *n* = 391		34 (9%)	90 (23%)	267 (68%)			
Birth order (391)	1	9 (5%)	27 (15%)	148 (80%)	1.0	1.0	1.0
	2	19 (15%)	47 (37%)	62 (48%)	4.22 (2.58–6.91)	4.23 (2.56–6.97)	4.83 (2.68–8.70)
	3+	6 (8%)	16 (20%)	57 (72%)	1.60 (0.87–2.93)	1.55 (0.83–2.87)	2.24 (0.92–5.49)
Total household members at 12 m (389)	median IQR, range	4 3–4, 2–5	4 3–4, 2–8	4 3–4, 2–8	1.06 (0.87–1.28)	1.06 (0.87–1.28)	0.89 (0.66–1.20)
No. of children (<16y) in household at 12 m (369)	median IQR, range	2 1–2, 1–4	2 1–2, 1–6	1 1–2, 1–5	1.26 (1.00–1.58)		-
Duration of breastfeeding (390)	never	8 (8%)	21 (21%)	73 (72%)	1.0	1.0	1.0
	<=4 weeks	4 (4%)	21 (21%)	74 (75%)	0.82 (0.44–1.52)	0.83 (0.44–1.56)	0.88 (0.45–1.72)
	1–6 months	11 (12%)	22 (23%)	62 (65%)	1.37 (0.76–2.49)	1.45 (0.79–2.68)	1.52 (0.78–2.95)
	6–12 months	8 (12%)	20 (31%)	37 (57%)	1.87 (0.99–3.53)	1.98 (1.04–3.79)	2.05 (0.98–4.31)
	>12 months	3 (10%)	6 (21%)	20 (69%)	1.16 (0.48–2.82)	1.20 (0.49–2.94)	0.99 (0.38–2.56)
Bedroom sharing in first year (389)	no	21 (9%)	50 (23%)	149 (68%)	1.0		-
	yes	13 (8%)	38 (22%)	118 (70%)	0.89 (0.58–1.37)		-
Bedroom sharing in second year (391)	No	20 (8%)	54 (22%)	174 (70%)	1.0	1.0	1.0
	yes	14 (10%)	36 (25%)	93 (65%)	1.26 (0.82–1.94)	1.25 (0.81–1.93)	1.11 (0.66–1.85)
Bed sharing in second year (391)	No	31 (8%)	83 (23%)	250 (69%)	1.0		-
	yes	3 (11%)	7 (26%)	17 (63%)	1.30 (0.59–2.87)		-
Formal child care up to 12 m visit (total hours) (381)	No childcare	13 (8%)	35 (22%)	113 (70%)	1.0	1.0	1.0
	<1165 total hours	2 (6%)	9 (26%)	23 (68%)	1.08 (0.49–2.35)	1.08 (0.49–2.36)	0.94 (0.40–2.19)
	> = 1165 total hours	14 (15%)	28 (29%)	53 (56%)	1.90 (0.13–3.18)	1.90 (1.13–3.18)	1.69 (0.92–3.11)
	No formal (only informal)	4 (4%)	16 (18%)	71 (78%)	0.65 (0.36–1.18)	0.65 (0.36–1.19)	0.66 (0.34–1.26)
Mother and baby activities in first year (389)	Rarely	16 (7%)	44 (21%)	149 (71%)	1.0		-
	At least once a month	18 (10%)	44 (24%)	118 (66%)	1.31 (0.86–2.00)		-
Mother and baby activities in second year (389)	Rarely	18 (11%)	32 (20%)	108 (68%)	1.0	1.0	1.0
	At least once a month	16 (7%)	57 (25%)	158 (68%)	0.94 (0.61–1.44)	0.91 (0.59–1.41)	0.75 (0.46–1.23)
Age 24 m blood sample taken (mths) (391)	23.59–24.99	7 (8%)	21 (25%)	56 (67%)	1.0	1.0	1.0
	25–26.99	20 (9%)	49 (21%)	159 (70%)	0.89 (0.52–1.50)	0.87 (0.51–1.48)	0.68 (0.38–1.21)
	27–32.89	7 (9%)	20 (25%)	52 (66%)	1.04 (0.55–1.97)	1.01 (0.53–1.92)	0.73 (0.37–1.47)
Pakistani		by 12 mths	12–24 mths	uninfected at 24 mths	Unadjusted OR (95% CI)	Unadjusted OR (95% CI) *n* = 466	Adjusted OR (95% CI) *n* = 466
All children, *n* = 472		46 (10%)	65 (14%)	361 (76%)			
Birth order (472)	1	5 (4%)	12 (10%)	109 (87%)	1.0	1.0	1.0
	2	13 (12%)	22 (21%)	72 (67%)	3.06 (1.61–5.83)	3.07 (1.61–5.86)	3.75 (1.89–7.43)
	3+	28 (12%)	31 (13%)	180 (75%)	2.17 (1.21–3.91)	2.16 (1.20–3.89)	3.05 (1.53–6.09)
Total household members at 12 m (468)	median IQR, range	6 5–7, 4–13	5 4–7, 3–11	6 5–7, 2–15	0.99 (0.90–1.09)	0.99 (0.90–1.09)	0.98 (0.88–1.09)
No. of children (<16y) in household at 12 m (456)	median IQR, range	3 2–3, 1–5	3 2–4, 1–6	3 2–4, 1–8	1.06 (0.91–1.25)	-	-
Duration of breastfeeding (468)	never	12 (14%)	11 (13%)	61 (73%)	1.0	1.0	1.0
	<=4 weeks	6 (7%)	11 (12%)	74 (81%)	0.58 (0.29–1.18)	0.59 (0.29–1.20)	0.64 (0.31–1.35)
	1–6 months	11 (9%)	15 (12%)	95 (79%)	0.70 (0.37–1.34)	0.70 (0.37–1.34)	0.86 (0.44–1.66)
	6–12 months	10 (9%)	16 (16%)	76 (75%)	0.87 (0.45–1.65)	0.88 (0.46–1.68)	1.03 (0.52–2.02)
	>12 months	7 (10%)	11 (16%)	52 (74%)	0.88 (0.43–1.79)	0.88 (0.43–1.79)	0.97 (0.47–2.02)
Bedroom sharing in first year (468)	no	2 (6%)	3 (10%)	26 (84%)	1.0	-	-
	yes	44 (10%)	61 (14%)	332 (76%)	1.64 (0.62–4.37)		-
Bedroom sharing in second year (472)	no	3 (13%)	2 (8%)	19 (79%)	1.0	1.0	1.0
	yes	43 (10%)	63 (14%)	342 (76%)	1.12 (0.41–3.05)	1.12 (0.41–3.07)	0.96 (0.33–2.79)
Bed sharing in second year (471)	No	26 (10%)	35 (13%)	206 (77%)	1.0		-
	yes	20 (10%)	30 (15%)	154 (75%)	1.09 (0.71–1.66)		-
Any regular child care arrangement up to 24 m visit (472)	no	40 (11%)	54 (14%)	286 (75%)	1.0	1.0	1.0
	yes	6 (7%)	11 (12%)	75 (81%)	0.68 (0.38–1.21)	0.64 (0.36–1.16)	0.78 (0.42–1.44)
Mother and baby activities in first year (468)	Rarely	42 (10%)	56 (14%)	307 (76%)	1.0		-
	At least once a month	4 (6%)	8 (13%)	51 (81%)	0.72 (0.37–1.40)		-
Mother and baby activities in second year (472)	Rarely	23 (9%)	30 (12%)	200 (79%)	1.0	1.0	1.0
	At least once a month	23 (10%)	35 (16%)	161 (74%)	1.34 (0.88–2.04)	1.37 (0.90–2.10)	1.43 (0.92–2.21)
Maternal age at recruitment (472)	Mean, SD, range	28.0, 4.48 19–39	28.8, 5.23 19–41	28.9, 5.38 17–44	0.98 (0.95–1.02)	0.98 (0.94–1.02)	0.95 (0.90–0.99)
Age 24 m blood sample taken (mths) (472)	23.59–24.99	7 (8%)	12 (13%)	72 (79%)	1.0	1.0	1.0
	25–26.99	20 (8%)	35 (15%)	185 (77%)	1.12 (0.63–2.01)	1.08 (0.60–1.94)	1.13 (0.62–2.08)
	27–32.89	19 (13%)	18 (13%)	104 (74%)	1.41 (0.75–2.63)	1.39 (0.75–2.61)	1.42 (0.74–2.73)

*VZV* varicella zoster virus; *OR* odds ratio; *CI* confidence interval; *IQR* inter-quartile range

## Discussion

One-third of Pakistani children were CMV infected in the first year compared to 9% of White British children. By 2 years, half of Pakistani children were EBV infected compared to a quarter of White British children. The higher incidence of CMV and EBV infection among Pakistani children cannot be fully explained by differences in breastfeeding, family size, childcare and socio-economic indicators. VZV incidence from 12 to 24 months was lower among the Pakistani than White British children. Risk factors for CMV and EBV infection differed by ethnic group.

Our VZV estimates are similar to those from previous UK studies [18, 24]. In the Millennium Cohort Study, children from other ethnic groups were less likely to have had chickenpox by age 3 years than white children, based on maternal report, similar to our findings [18]. There is good evidence that VZV seroprevalence in the UK is lower among pregnant women born in Asia than those born in the UK [13, 25], due to later age at infection in many tropical countries compared to the UK. However, it is unclear why children born to Asian women in the UK are infected later, as VZV is predominantly transmitted between children rather than from contact with the mother. It has been suggested that infection with CMV affords some cross-protection against VZV [18, 26] which could explain the later VZV infection in Pakistani children in our study as they are infected with CMV earlier than the White British children.

Our CMV incidence results were similar to a cross-sectional study in the Netherlands [27]. CMV seroprevalence in 1- and 2-year-olds in a US study was lower than our overall results, although there were small numbers of children in each age group (<200) [11]. The only other UK study on CMV prevalence in young children was based in a London hospital 30 years ago and 20% of children were infected by 12 months [16]. Results from Australia and Sweden showed slightly higher seroprevalence at 2 years [28, 29]. Our CMV results on breastfeeding, childcare and socio-economic indicators are consistent with previous findings and we report novel results on birth order (Table 5).

**Table 5:** Summary of main associations with age at infection

		CMV	EBV	VZV
Associated with earlier infection	White British	• household size^c^ • breastfeeding >6m^b^ [16, 32, 42] • formal childcare in 1st yr^b^ [32] • not owner-occupied household^b^ [11]	• informal childcare <1165 hrs^c^	• birth order^b^ [18]
	Pakistani	• breastfeeding^b^ • mother born outside UK/Ireland ^c^	• bedroom sharing in 1st yr^b^ [21]	• birth order^b^
Associated with later infection	White British	-	• mother/baby activities in 1st yr^a^	-
	Pakistani	• birth order^a^ • travel outside Europe in 1st yr*^b^	-	• maternal age^a^

^a^novel associations

^b^previous studies with similar findings (refs)

^c^expected association

*assuming this reflects higher socio-economic position

Ethnicity was a key risk factor in several studies with consistent findings of higher CMV prevalence among children in non-white ethnic groups compared to white/European/Western groups [11, 27, 30]. This association was specifically investigated in relation to other factors in studies in New Zealand [31] and the Netherlands [32] in children aged 3.5 and 6 years, respectively. Differences by ethnic group could not be explained by differences in parity, day care, breastfeeding, and socio-economic indicators, similar to our findings.

The unexpected apparent protective effect of higher birth order on early CMV infection among Pakistani children is unlikely to be spurious for several reasons. First, the birth order effect commonly associated with childhood infections (i.e. children of higher birth order more likely to be infected earlier) was observed for VZV in our study. Second, estimates for the White British children are also less than one although the confidence intervals cross one. Third, Jansen et al. report a similar finding; multiparity was inversely associated with CMV seropositivity by age 6 years (OR 0.76, 95% CI 0.65–0.90) [32]. The ‘birth order effect’ is usually discussed in relation to the hygiene hypothesis and refers to the lower risk of atopic disease among children of higher birth order due to increased exposure to childhood infections [15]. An alternative or contributing explanation for this effect could be changes in the maternal immune system with increasing parity so that the effect is determined in the prenatal period [33]. Whether these changes could also affect the maternal immune response to CMV infection and thereby the risk of transmitting virus to her child (e.g. through breastmilk) warrants further investigation. Perhaps CMV infection in the first child acts like a vaccine to boost maternal immunity so she has lower viral load and takes longer to infect subsequent children. Any such effect might be manifest through acquiring immune responses to strains of CMV different from the initial maternal infection, because prototype CMV vaccines can boost the immunity of seropositive adults [8, 34, 35].

Our EBV incidence estimates are comparable to results from previous UK studies [36–38]. Differences in paediatric EBV prevalence by ethnic group have been reported from two US studies [22, 39] and one in the Netherlands [32]. Socio-economic position and family size partly explained the ethnic differences observed in the Generation R study [32] but the lower prevalence in white children could not be explained by infection risk factors in the Minnesota study [39].

The unexpected association between attendance at mother/baby activities and later EBV infection among White British children may have arisen due to uncontrolled confounding by socio-economic factors; further investigation did not provide convincing evidence of a real effect as the association was only evident in a sub-group.

Ethnic differences in paediatric CMV and EBV incidence have not been reported before in the UK but our results are in line with findings in different populations. As these cannot be fully explained by factors influencing exposure to infection, genetic variation may play a role. For example, the prevalence of the NKG2C deletion is known to vary between populations and is important in NK cell responses to CMV infection such that individuals with the deletion are less able to control CMV viraemia so CMV may be more easily transmitted [40]. Genetic susceptibility may also be important in infectious mononucleosis following EBV infection [41].

A key strength of this study is having serology data at two time points early in childhood. Previous studies have been cross-sectional or with samples available for only one time point. Our results demonstrate that most of the CMV infections occurred in the first year and are generally due to contact with the mother, whereas most EBV and VZV infections are acquired in the second year through contact with siblings and other children. Pinpointing age at infection is important when examining associations with subsequent development of atopic disease [42, 43] as there is likely to be a critical window in immune development in the first 2 years during which infection with CMV and/or EBV may influence immune-related outcomes.

We collected detailed information on likely sources of exposure to infection, with few missing values due to interviewer-administered questionnaires with well-trained interviewers. This study had the statistical power to investigate differences in acquisition of infection between two large ethnic groups and the advantages of a prospective birth cohort design with rich characterisation and homogeneity within ethnic groups. There were few differences between the children in this analysis and the main BiB cohort and our results are generalisable to other multi-ethnic urban UK populations. Our results may not be generalisable to less deprived populations within the UK where the incidence of CMV and EBV infection by age 2 years is likely to be lower than in Bradford.

A limitation of our study is that maternal CMV serostatus was unavailable for most children. Adjustment for mother’s country of birth and maternal age would have partially accounted for maternal serostatus as South Asian women born abroad have higher seroprevalence than those born in the UK [13] and seroprevalence increases with age. The pattern of higher incidence at 12 months in Pakistani than White British children was still evident in the sub-group of CMV seropositive women.

Misclassification of age at CMV and VZV infection due to the occasional presence of maternal antibody at 12 months is likely to have had a minimal effect; the adjusted incidence figures rounded to the same percentage points.

## Conclusions

This study provides current incidence data for three common herpesviruses in young children which are generalisable to urban UK populations. We have demonstrated large differences in incidence between White British and Pakistani children for the first time in the UK and have identified key risk factors for infection in each group, some novel, which suggest mechanisms of transmission to be investigated further.

Our data will inform the optimum schedule of potential CMV and EBV vaccination programmes. For CMV it has been proposed that toddlers as well as adolescents are vaccinated to prevent transmission to pregnant women and reduce the risk of congenital CMV infection [9]. Our results suggest that infants of Pakistani origin would need to be vaccinated before age one and the impact of vaccinating children with recent natural infection needs to be explored.

## Additional files

Additional file 1: Further details of Methods; text giving further details of the Methods including sample size calculations. (DOCX 12 kb)
Additional file 2: Total hours of informal and formal childcare by ethnic group; table showing categories of total hours of informal and formal childcare by ethnic group. (DOCX 13 kb)
Additional file 3: CMV, EBV and VZV incidence data for Figure 2; table showing the CMV, EBV and VZV incidence data presented in Figure 2. (DOCX 13 kb)

## Abbreviations

ALL IN: Allergy and Infection Study
BiB: Born in Bradford
CI: Confidence interval
CMV: Cytomegalovirus
DNA: Deoxyribonucleic acid
EBV: Epstein Barr virus
IgG: Immunoglobulin G
OR: Odds ratio
VZV: Varicella-zoster virus
