# Supplementary material for: Cytomegalovirus, Epstein-Barr virus and varicella zoster virus infection in the first two years of life: a cohort study in Bradford, UK
Source: BMC Infect Dis. 2017 Mar 21;17:220. doi: 10.1186/s12879-017-2319-7 (PMC5360071; doi:10.1186/s12879-017-2319-7)
Supplement: Additional file 1: — Further details of Methods; text giving further details of the Methods including sample size calculations. (DOCX 12 kb) [file 12879_2017_2319_MOESM1_ESM.docx]

**Additional File 1**

**Further details of Methods**

Community research administrators (CRAs) received training in phlebotomy and were assessed by the senior paediatric phlebotomist at Bradford Royal Infirmary. Ametop cream or Cryogesic spray were used to anaesthetise the venepuncture site. Only two attempts at venepuncture were permitted for each child. CRAs were also trained in obtaining informed consent and administering the ALL IN questionnaires. Numerous attempts to contact parents were made to ensure the largest response rate possible. The use of text messages and evening phone calls were introduced during the study to increase recruitment. If the family were not at home at the arranged time or did not attend their clinic appointment, they were contacted again to arrange another appointment and at least two further attempts to complete a visit were made. A study database (MS Access) was used to record contacts with parents and visits attempted and completed for each child.

*Sample size*

The sample size was based on identifying risk factors for infection; 852 was the largest sample size required to detect a 1.5-fold difference in prevalence of CMV infection at 12 months between breastfed and non-breastfed babies with 80% power and 5% significance, assuming that 75% of babies are breastfed and 20% of infants are CMV infected by 12 months, and to allow for multivariable analyses.
