# Supplementary material for: Cytomegalovirus, Epstein-Barr virus and varicella zoster virus infection in the first two years of life: a cohort study in Bradford, UK
Source: BMC Infect Dis. 2017 Mar 21;17:220. doi: 10.1186/s12879-017-2319-7 (PMC5360071; doi:10.1186/s12879-017-2319-7)
Supplement: Additional file 2: — Total hours of informal and formal childcare by ethnic group; table showing categories of total hours of informal and formal childcare by ethnic group. (DOCX 13 kb) [file 12879_2017_2319_MOESM2_ESM.docx]

|  | **n** | **CMV** | | **EBV** | | **VZV** | |
| --- | --- | --- | --- | --- | --- | --- | --- |
|  |  | **12 months** | **24 months** | **12 months** | **24 months** | **12 months** | **24 months** |
| White British | 391 | 9% (36) | 15% (61) | 7% (28) | 25% (99) | 9% (34) | 32% (124) |
| Pakistani | 472 | 34% (162) | 44% (209) | 21% (101) | 51% (241) | 10% (46) | 23% (110) |
| Indian or Bangladeshi | 57 | 32% (18) | 42% (24) | 11% (6) | 39% (22) | 7% (4) | 14% (8) |
| other | 80 | 19% (15) | 30% (24) | 10% (8) | 40% (32) | 3% (2) | 18% (14) |
| All | 1000 | 23% (231) | 32% (318) | 14% (143) | 39% (394) | 9% (86) | 26% (256) |

**Additional File 2. Table: CMV, EBV and VZV incidence data for Figure 2**
