# Supplementary material for: Cytomegalovirus, Epstein-Barr virus and varicella zoster virus infection in the first two years of life: a cohort study in Bradford, UK
Source: BMC Infect Dis. 2017 Mar 21;17:220. doi: 10.1186/s12879-017-2319-7 (PMC5360071; doi:10.1186/s12879-017-2319-7)
Supplement: Additional file 3: — CMV, EBV and VZV incidence data for Figure 2; table showing the CMV, EBV and VZV incidence data presented in Figure 2. (DOCX 13 kb) [file 12879_2017_2319_MOESM3_ESM.docx]

**Additional File 3. Table: Total hours of informal and formal childcare by ethnic group**

|  | All  (n=1000) | White British (n=391) | Pakistani  (n=472) |
| --- | --- | --- | --- |
| **Informal child care up to 12m visit** |  |  |  |
| No childcare | 646 (67%) | 161 (43%) | 405 (88%) |
| <1165 total hours | 178 (18%) | 119 (32%) | 33 (7%) |
| >=1165 total hours | 25 (3%) | 15 (4%) | 5 (1%) |
| No informal (only formal) | 117 (12%) | 81 (21%) | 16 (3%) |
| Hours missing for informal | 28 | 13 | 9 |
| **Formal child care up to 12m visit** |  |  |  |
| No childcare | 646 (66%) | 161 (42%) | 405 (87%) |
| <1165 total hours | 43 (4%) | 34 (9%) | 2 |
| >=1165 total hours | 129 (13%) | 95 (25%) | 16 (3%) |
| No formal (only informal) | 165 (17%) | 91 (24%) | 45 (10%) |
| Hours missing for formal | 11 | 8 | 0 |

| **Informal child care from 12m visit to 24m visit** |  |  |  |
| --- | --- | --- | --- |
| No childcare | 574 (58%) | 125 (33%) | 380 (81%) |
| <1165 total hours | 57 (6%) | 44 (11%) | 9 (2%) |
| >=1165 total hours | 67 (7%) | 41 (11%) | 15 (3%) |
| No informal (only formal) | 164 (16%) | 107 (28%) | 31 (7%) |
| No 24m childcare, only 12m | 127 (13%) | 67 (17%) | 35 (7%) |
| Hours missing for informal | 11 | 7 | 2 |
| **Formal child care from 12m visit to 24m visit** |  |  |  |
| No childcare | 574 (57%) | 125 (32%) | 380 (81%) |
| <1165 total hours | 30 (3%) | 23 (6%) | 6 (1%) |
| >=1165 total hours | 185 (19%) | 127 (32%) | 30 (6%) |
| No formal (only informal) | 82 (8%) | 49 (13%) | 20 (4%) |
| No 24m childcare, only 12m | 127 (13%) | 67 (17%) | 35 (7%) |
| Hours missing for formal | 2 | 0 | 1 |
